# Structural analyses in the study of behavior: From rodents to non-human primates

**DOI:** 10.3389/fpsyg.2022.1033561

**Published:** 2022-11-18

**Authors:** Maurizio Casarrubea, Jean-Baptiste Leca, Noëlle Gunst, Gudberg K. Jonsson, Mariona Portell, Giuseppe Di Giovanni, Stefania Aiello, Giuseppe Crescimanno

**Affiliations:** ^1^Laboratory of Behavioral Physiology, Department of Biomedicine, Neuroscience and Advanced Diagnostics (Bi.N.D.), Human Physiology Section “Giuseppe Pagano”, University of Palermo, Palermo, Italy; ^2^Department of Psychology, University of Lethbridge, Lethbridge, AB, Canada; ^3^School of Natural and Engineering Sciences, National Institute of Advanced Studies, Bangalore, India; ^4^Human Behavior Laboratory, School of Health Sciences, University of Iceland, Reykjavík, Iceland; ^5^Department of Psychobiology and Methodology of Health Sciences, Universitat Autònoma de Barcelona, Cerdanyola del Vallès, Spain; ^6^Laboratory of Neurophysiology, Department of Physiology and Biochemistry, Faculty of Medicine and Surgery, University of Malta, Msida, Malta

**Keywords:** hierarchical clustering, transition probabilities, adjusted residuals, T-pattern analysis, behavioral structure–function interface

## Abstract

The term “*structure*” indicates a set of components that, in relation to each other, shape an organic complex. Such a complex takes on essential connotations of functionally unitary entity resulting from the mutual relationships of its constituent elements. In a broader sense, we can use the word “*structure*” to define the set of relationships among the elements of an emergent system that is not determined by the mere algebraic sum of these elements, but by the interdependence relationships of these components from which the function of the entire structure itself derives. The behavior of an integrated living being can be described in structural terms *via* an ethogram, defined as an itemized list of behavioral units. Akin to an architectural structure, a *behavioral structure* arises from the reciprocal relationships that the individual units of behavior establish. Like an architectural structure, the *function* of the resulting behaving complex emerges from the relationships of the parts. Hence, studying behavior in its wholeness necessitates not only the identification of its constitutive units in their autarchic individuality, but also, and importantly, some understanding of their relationships. This paper aimed to critically review different methods to study behavior in structural terms. First, we emphasized the utilization of T-pattern analysis, i.e., one of the most effective and reliable tools to provide structural information on behavior. Second, we discussed the application of other methodological approaches that are based on the analysis of transition matrices, such as hierarchical clustering, stochastic analyses, and adjusted residuals. Unlike T-pattern analysis, these methods allow researchers to explore behavioral structure beyond its temporal characteristics and through other relational constraints. After an overview of how these methods are used in the study of animal behavior, from rodents to non-human primates, we discussed the specificities, advantages and challenges of each approach. This paper could represent a useful background for all scientists who intend to study behavior both quantitatively and structurally, that is in terms of the reciprocal relationships that the various units of a given behavioral repertoire normally weave together.

## Introduction

“*The ability to reduce everything to simple fundamental laws does not imply the ability to start from those laws and reconstruct the universe*.”

Anderson (1972)

This paper deals with behavior and with a number of approaches useful to study it. We asked: “*what* is the behavior of a living being?” Such a question, simple and straightforward, cannot be propaedeutic to an answer characterized by the same features. Using a common scientific database and searching the term “*behavior*” produce thousands of definitions, explanations, points of view, theories etc. Some definitions are very cryptic, others fanciful, others difficult to understand and others more or less agreeable. Not surprisingly, some of the strongest definitions of behavior were proposed by the founding Figures of Ethology such as Konrad Lorenz, Nikolaas Tinbergen or Irenäus Eibl-Eibesfeldt: behavior is the reaction of a living being to external (Tinbergen, 1951a) or internal (Tinbergen, 1951b) *causal factors* and it is *organized* on the basis of a *sequence* of events in *time* (Eibl-Eibesfeldt, 1970). These words echo a central dogma in ethology and in most disciplines belonging to behavioral sciences. Indeed, they relate to the causality and temporal organization of behavior. One thing is self-evident: like its definition, the methods to study behavior are not simple and straightforward.

### Structures, functions, and processes

In his well-known writing titled “Principles of the self-organizing system,” the brilliant English psychiatrist William Ross Ashby stated that if the relationship between two entities, e.g., “A” and “B,” becomes conditional on “C,” then a necessary component of organization is present and, as a result, a whole composed of interacting parts (i.e., a structure) emerges (Ashby, 1962). Closely related to the concept of “structure” is the dual and dynamic relationship between function and process. The concept of “*function*” is permeated with purely finalistic, i.e., teleological, connotations. Thus, the word “*function*” denotes the purpose of the object “*sensu lato*,” whether it is a very simple or extremely complex item. Two common questions, that is “*what is it for?*” and “*what is its purpose?*,” perfectly express the teleological nature of any object. What is the purpose of one of the many walls we can easily observe in a house? While trivial, this example gives a good understanding of the concept of teleology: it serves to separate two rooms, or an interior space from the exterior of the house. The wall, then, does create a division that makes possible a better use of two rooms that would otherwise be a single, much less usable space. However, this explanation says nothing about *how* the wall came to be. As much as the answer just given regarding the finalistic aspects is more or less articulated, in fact, we have left out the second and essential part of the above binomial: the processual component. In contrast to function, the concept of “*process*” is imbued with purely mechanistic connotations. The term “*process*” therefore indicates *how* something happens. The fundamental question related to it is no longer “*what does it do*?” but “*how does it do it*?.” Returning to our example, then, we may say that a wall, capable of performing the above functions, consists of the juxtaposition of bricks, with a specific orientation within the apartment, properly joined together thanks to a specific amalgam that prevents displacements between neighboring bricks and gives greater stability to the structure. As will be noted, these purely mechanistic aspects complement and explicate the question about the above purpose.

Even the unexperienced reader will not miss the enormous difference between the concepts of “*function*” and “*process*” and their very close connection with the structure generating them. All of this, while of wide application in numerous fields (such as the example of wall and bricks), finds extraordinary congruity, in the biological realm, with one discipline in particular. The concepts of “function” and “process” permeate Physiology to the extent that they constitute the deeper and essential “*raison d’être*” of this entire discipline. In Physiology, these two concepts are the two sides of the same coin. The examples are potentially endless and all, conceptually, similar to the example of the bricks and the wall. For instance, when studying the heart, physiologists typically describe the various properties of this extraordinary organ, pointing out how its prodigious morphological characteristics (i.e., structure) enable it to constantly and tirelessly feed blood into the aorta and pulmonary vessels (i.e., functions) through specific mechanisms involving both electrical and purely mechanical phenomena (i.e., processes). Everything in the domain of Physiology orbits around functions and processes, and arguably, there is no system, organ, tissue and/or even single cell, able to escape such a binomial. Studying Physiology means, therefore, being able to describe with absolute precision each of the two sides of this coin: the functions and processes, the *why* and the *how*. These concepts apply to the study of behavior for the reasons discussed below.

Detailed knowledge about behavioral structure contributes to testing hypotheses about behavioral function. The “design-feature argument” holds that thorough structural analysis of a given behavior pattern provides valid information about its hypothesized function (Martin and Caro, 1985; Moran, 1985). The heuristic power of this behavioral structure–function interface is reflected in the following statement by Pellis and Pellis (1998): “*Therefore, behavioral description informs functional inference, which in turn, influences further description*” (p. 115). Thus, broad similarities and subtle differences in the structural organization of evolutionarily related behaviors are indicative of their respective motivational underpinnings and functional features. This approach has proved particularly useful to compare pairs of behavioral traits that are developmentally and evolutionary linked, but vary in their functional constraints. For example, among different types of object manipulation in non-human primates and pre-school children, researchers used structural variables, either based on kinematic or temporal components, to infer underlying psychological mechanisms and explain the actions being performed in terms of relative purpose and utility (e.g., object play and object exploration: Hughes, 1978, 1979; percussive object play and extractive foraging: Pelletier et al., 2017; Pellis et al., 2019). These studies indicate that utilitarian motivational processes and functional constraints in object manipulation covary with behavioral structure. When comparing two types of manipulative activities, the more product-oriented one (e.g., object exploration, extractive foraging) show higher levels of kinematic and/or temporal structure than the more process-oriented one (e.g., object play; see also Rasa, 1984).

In line with one of the basic tenets of Darwinian evolution, holding that selection strength and phenotypic variability are negatively correlated, more functionally constrained behaviors are subjected to higher selection pressures, which in turn, lead to less structurally variable behaviors. Thus, the behavioral structure of object-directed activities may be used as a proxy to assess their relative functionality. Theoretically, this powerful structure–function interface could be applied to other pairs of behavioral traits that are linked at the proximate levels (i.e., in their developmental trajectories and underlying sensori-motor and cognitive mechanisms) and at the ultimate levels (i.e., in their functional consequences and phylogenetic pathways; Tinbergen, 1963). Among other pairs of behavioral traits that were subjected to differential functional constraints and whose putative mechanistic and evolutionary connections are unravelled by in-depth structural analysis, let us mention object play and tool use (Cenni et al., 2020, 2022) as well as female-to-female mounting and female-to-male mounting (Gunst et al. 2020, 2022).

### Physiology of behavior

**Figure 1:**
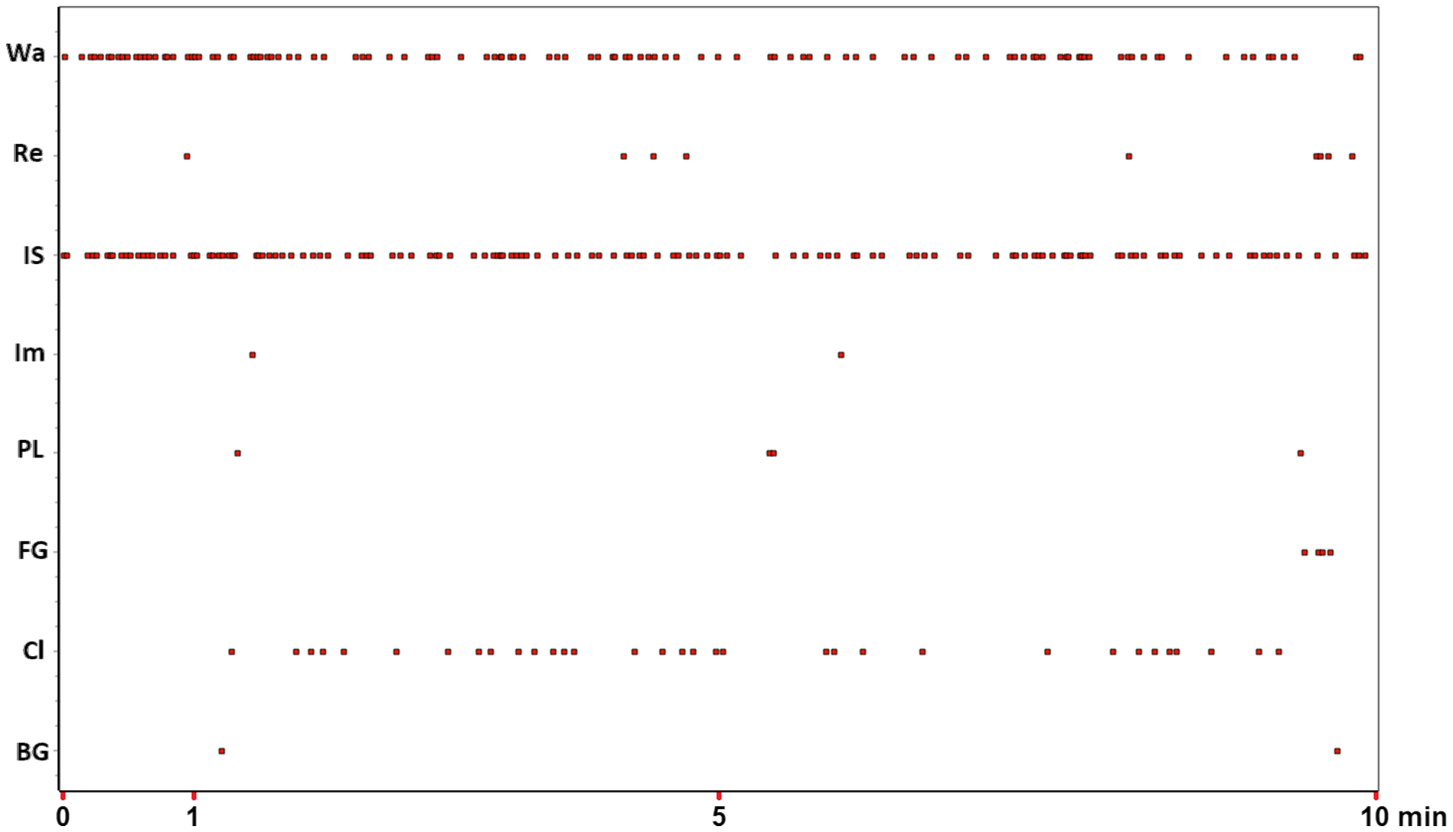
Event plot of 8 behavioral units (Y-axis) occurring during a 10-min (X-axis) observation window. For abbreviations of behavioral units on Y-axis see Table 1. Data from one subject taken from Casarrubea et al. (2020b). See text for details.

Behavior, whether of an insect or a primate, can be broken down into simpler units, each indicative of a specific and characteristic part of the subject’s behavioral repertoire. Imagine a fruit fly flying in a room, a rat moving through an Open Field, a non-human primate exploring a new part of a forest. Ethologists typically divide each of these activities into several behavioral sub-units, use simple and unambiguous names to label them, and generate operational definitions to describe them, thereby constructing an “*ethogram*.” For example, the behavior of a rodent may be referred to as “Walking” when the animal moves relatively slowly and quadrupedally from one quadrant of the arena to another, “Climbing” when its upper body leans against the perimeter walls, “Rearing” when it lifts up on its hind legs without leaning against any of the nearby walls, “Face Grooming” or “Body Grooming” when it picks at or scratches its head or other body parts, respectively, with its paws or mouth, etc. Identifying each of these behavioral units is a relatively simple task that only requires a fairly short training period. Practically, the observer watches the video of the animal’s behavior and, through a specific computer program, e.g., The Observer software coder (Noldus Information Technology, Netherlands), scores the occurrences of each behavioral unit. The product of this simple, but terribly time-consuming, process is called *event log-file* and consists of a generally long list of the behavioral events performed by the animal with their respective onset times. An event log-file can be conveniently illustrated by means of an event plot. Figure 1 is an example of an event plot obtained from one, specific pathogen-free, 2-month-old male C57/BL wild-type mouse (Jackson Laboratories, United States), belonging to a group of subjects, observed in the open-field for 10 min (Casarrubea et al., 2020b). A simple question arises: after watching 10 min of this video, after carefully observing the rodent’s exploratory behavior through the open field, after recognizing hundreds of Walking, Climbing, Rearing, Face Grooming etc., what to do with these data? Figure 1 shows more than 300 behavioral units. In abscissa we report the time, in ordinate the behavioral unit performed. The plot highlights where each behavioral unit falls in time. Walking occurred 114 times, Rearing 9 times, Immobile-Sniffing 136 times etc. Table 1 shows the name, frequency, percentage and overall duration of the behavioral units plotted in Figure 1. This table can be enriched with numerous additional quantitative evaluations such as, for example, the average duration of each behavior, the latency of the first appearance of each behavior etc.; one may also do somewhat more complex analysis such as, for example, evaluating the average duration of each item in relation to the time of observation, so as to provide a time course for each minute of observation. The first evidence, even with a very first glance of Table 1, is the remarkable feeling of completeness and comprehensiveness. The catch is, however, just around the corner. All these numbers *per se* tell us little about the complexity of an individual’s behavior. Taking into consideration our trivial example with the wall and the bricks, we argue that describing behavior in terms of individual units is like considering individual bricks and their many characteristics (i.e., weight, height, length, width etc.); however, it ignores the most important aspect, namely, what flows from their relationships both in terms of structure and the related functional and processual elements. As we previously noted, using a different and perhaps more effective metaphorical image, evaluating a subject’s behavior by considering only the individual units and not their relationships “[…] *is not different from classifying all the single pieces of a puzzle missing the comprehensive picture. The functional meaning of a behavior,* i.e.*, the study of the existing interplay between an animal and the context, is a picture lying in its intrinsic structural features*” (Casarrubea et al., 2019a). Actually, Figure 1 contains the bricks of our wall. Each dot in the plot highlights the specific “brick” shown on the Y axis and the time in which it occurred, shown on the X axis. Not all bricks are the same, just as the different types of adhesives we can use to build the given structure are not the same at all. However, if proper glue is not used between the individual units, they will not have solid reciprocal relationships and the final structure will not only be more unstable and/or difficult to build, but also prone to a rapid collapse. There is more. Not only we need the appropriate bricks and cement for the structure we want to build, the means we employ should also be adequate for the purpose. The question, then, is about where the meaning of behavior lies. Is it in the study of isolated behavioral units, each characterized by dozens of numbers, but still fragments of what is the animal’s true behavior? Or, conversely, is it in trying to relate all the behavioral units described in our observation in an attempt to reconstruct the true behavior? The answer is obvious. Studying behavior in its wholeness therefore means relating the individual units because only from these relationships arises the structure that is, in turn, preparatory to the functions and processes it serves and performs. In this lies the Physiology of Behavior. However, before delving into the different methods to study the relationships existing between the different units of the behavioral repertoire, it is important to clear the field from possible misunderstandings.

**Table 1:** Synoptic table summarizing occurrences, percent distribution and duration of behavioral units listed in the first column (abbreviations in brackets) and illustrated by means of event plot in Figure 1.

Component	Occurrences	Percent (%)	Duration (sec)
Walking (Wa)	114	37.50	181.57
Rearing (Re)	9	2.96	7.63
Immobile-sniffing (IS)	136	44.74	329.25
Immobility (Im)	2	0.65	2.75
Paw licking (PL)	4	1.32	8.67
Face grooming (FG)	4	1.32	11.7
Climbing (Cl)	33	10.86	50.93
Body grooming (BG)	2	0.65	7.5
Tot	304	100	600

Data from 1 subject taken from Casarrubea et al. (2020b). See text for details.

### Simple quantities and their usefulness

What we discussed in section “physiology of behavior” might lead to a seemingly logical but deeply flawed juxtaposition: one might believe that a purely quantitative approach is somewhat useless in the context of a behavioral study. We clearly do not support such a biased view. The meticulous description of each unit of behavior, albeit unconnected with the relational dynamics of the different units, can find numerous applications. If, for example, we look again at Table 1, it is certainly useful to know the more frequent and the less frequent behavior, or those that last longer and those that are shorter etc. Based on this information, it becomes possible to know what the subject does and, importantly, how long it takes to do it. In addition, one may examine if and how these items change following the administration of an independent variable such as the benzodiazepine Diazepam, a drug with anxiolytic action, that induces a conspicuous increase in Walking (Casarrubea et al., 2009b), or the administration of the beta-carboline FG7142, a potent anxiety inducing molecule, causing a significant decrease in Walking (Casarrubea et al., 2017). The explanation lies in the modulation of the state of anxiety of the two molecules: with reduced anxiety, the animal explores the environment more (and therefore walks more), whereas with increased anxiety the subject tends to move much more circumspectly (and therefore walks less). These examples highlight the usefulness of the descriptive/quantitative approach when dealing with discrete variables. However, in both cases, nothing can be said about what strategies the animal deploys, how it relates to the environment etc. In short, we still know little about exactly what the animal is doing and precisely how it is doing it; specifically, information about the pharmacological underpinnings of the structure of its behavior in its organic entirety remains scanty.

## Structural analyses in the study of behavior

Three prerequisites, propaedeutic to each other, are essential to study behavior through the relationships among the different units of the behavioral repertoire: one must (a) clarify what these relationships are, (b) have the appropriate means to study these relationships, (c) use these methods to tie together the different units of the behavioral repertoire before any discussion about behavioral structure can occur.

How do the different behaviors illustrated in Figure 1 and carefully described in Table 1 relate to each other? Are there constraints of any kind applied to them? If so, what methods can researchers use to describe such constraints? One way to assess the relationships between a behavior and the following one is to calculate reciprocal transition frequencies. Probabilistic constraints may also be evaluated by determining the probability of behavioral unit “A” shifting to behavioral unit “B.” One may also examine whether the transitions between these two units occur more or less frequently than an expected value. Furthermore, it is essential to consider what is certainly the most important relational constraint: the temporal one. Here the question becomes: Is it possible for different behavioral units to occur at fixed intervals or, at the very least, is it possible to identify specific temporal distances among different behaviors or only between some? Such temporal constraints may not be, and in fact rarely are, rigidly sequential.

All these techniques describing, and sometimes unravelling, relationships between units of the subject’s behavioral repertoire, belong to the realm of multivariate analyses. These methods, although not recently conceived and introduced in behavioral research, have seen a growing and steady diffusion only in recent decades, hand in hand with the development of, and easy access to, personal computers. Indeed, most of these analyses are very complex and involve considerable amounts of data, the management of which greatly benefits from the continuing growth of computational capabilities allowed by modern personal computers. In the following sections, we present some useful approaches to describing behavior in structural terms, that is, through the relationships among different units of the behavioral repertoire. These sections do not claim to be exhaustive regarding the totality of methods that can be employed to solve these problems. Rather, we present a number of approaches that, from our 30 years of experience in the field, have proven to be more than efficient and useful in this regard. These approaches can be divided into two major groups: methods based on the use of transition matrices and methods based on the use of T-pattern analysis.

### Transition matrices and related analyses

**Figure 2:**
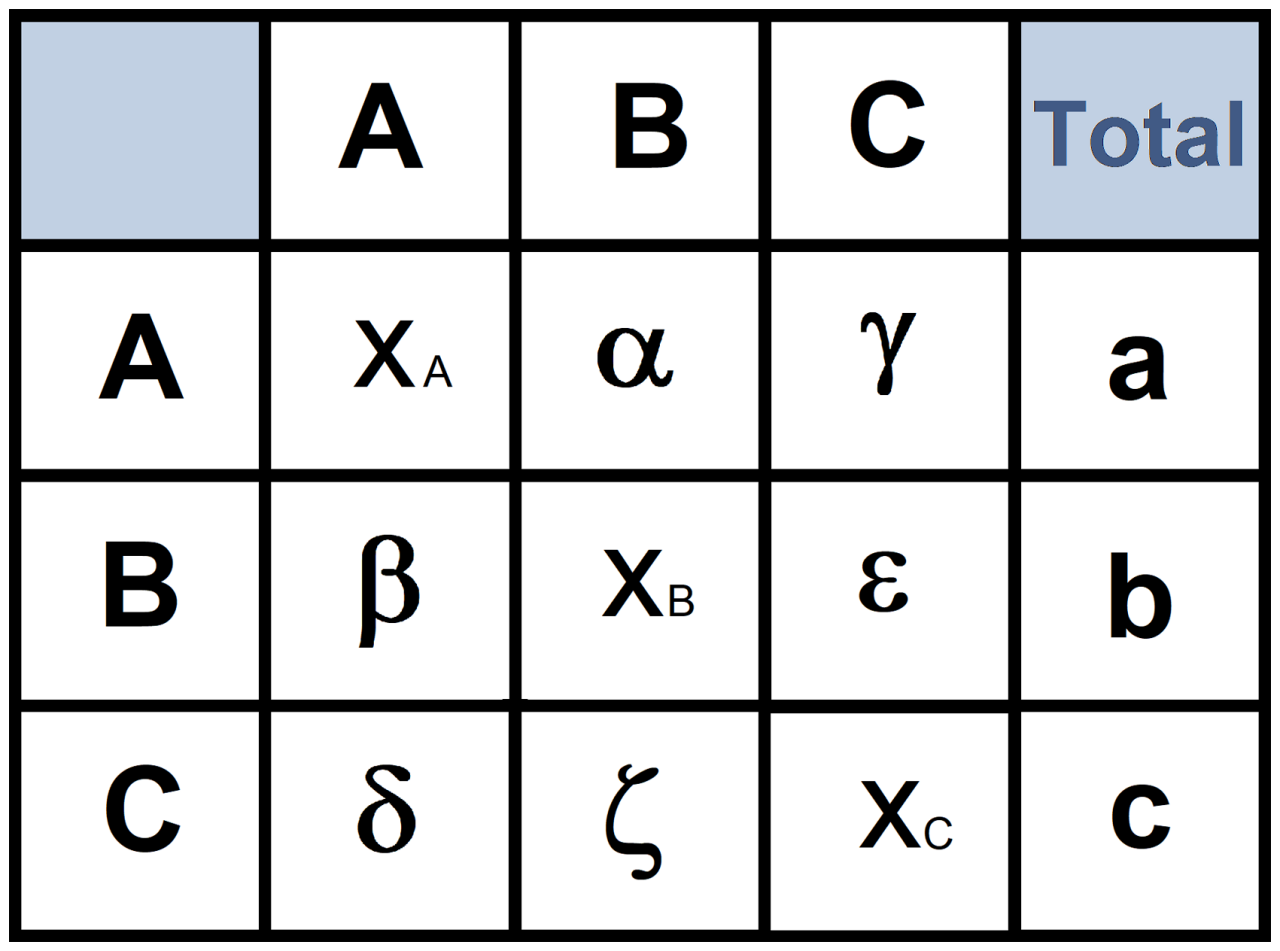
Example of transition matrix. A, B, C: names of three hypothetical behavioral units; α, number of transitions from unit A to B; γ: number of transitions from A to C; β: number of transitions from B to A; ε: number of transitions from B to C; δ: number of transitions from C to A; ζ: number of transitions from C to B; X_A_, X_B_ and X_C_: number of auto-transitions from A to A, B to B, and C to C, respectively; a: total occurrences of A; b: total occurrences of B; c: total occurrences of C.

The structure of a hypothetical transition matrix is illustrated in Figure 2. The utilization of transition matrices implies three requirements (1) the number of transitions contained in a transition matrix must be at least five times the number of units of the behavioral repertoire (Spruijt and Gispen, 1984); (2), the number of empty cells must be no more than 20% (Spruijt and Gispen, 1984; Casarrubea et al., 2008); and (3) the number of subjects used should be at least three times the number of behavioral units (Short and Horn, 1984; Espejo and Mir, 1993). These three requirements allow researchers to generate a number of transitions sufficient to avoid a poorly filled matrix (Spruijt and Gispen, 1984).

#### Hierarchical clustering

**Figure 3:**
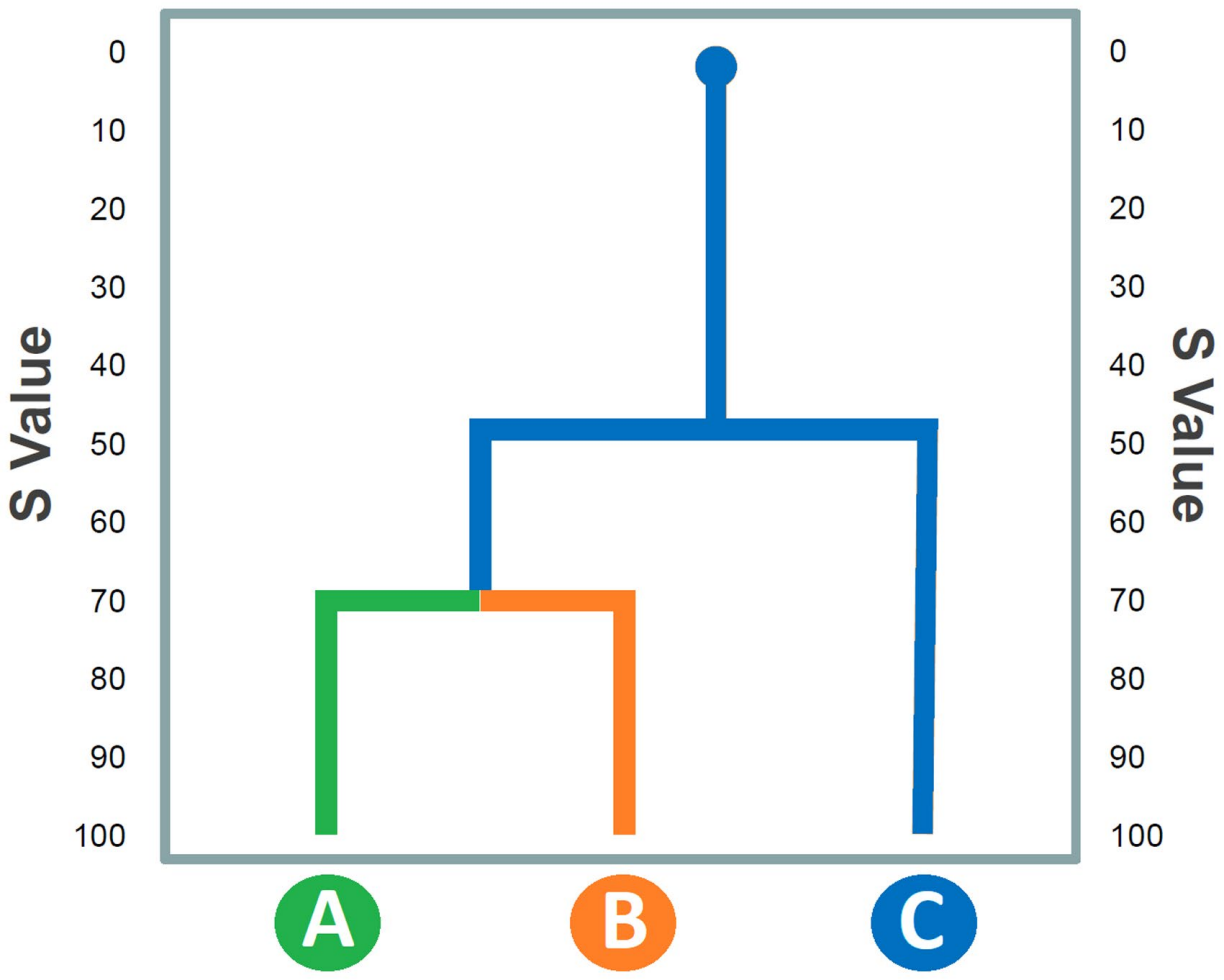
Hypothetical dendrogram representing similarity values (S: *Y*-axis) among behavioral units (*X*-axis) on the basis of a half matrix arranged following an aggregative clustering procedure. See text for details.

Cluster analysis aims to highlight constraints among units of the behavioral repertoire on the basis of the overall number of reciprocal transitions occurring during the overall observation period. To this purpose, a transition matrix must be transformed into a similarity matrix by using an aggregative procedure. Several aggregative procedures are available. For instance, in Figure 2, the similarity between the behavioral unit A and B can be obtained by using the procedure described by Mos et al. (1987):


S=(α/a+α/b+β/a+β/b)50

where S is the “similarity” value between units “A” and “B,” Greek letters “α” and “β” respectively indicate number of transitions from unit A to unit B and from unit B to A, lowercase letters “a” and “b” respectively indicate the total occurrences of A and B, and 50 is a normalization factor. This procedure must be repeated for each pair of cells within the matrix. The result is a half matrix where each cell does not express a transition but the “*vicinity*” between two variables (rows and columns names). This approach thus assumes that the number of transitions can be considered a valid index of aggregation between two or more elements, a premise that should be taken into serious consideration when interpreting the data. Indeed, talking about “*similarity*” value between two behavioral units might be misconstrued as these two units being qualitatively similar. In reality, the *closeness* expresses a high number of mutual transitions, not a phenomenological similarity at all. For example, in rats, Edge-Sniff behavior (i.e., when the animal sniffs the edge of the hole in a test called “Hole-Board”) and Head-dip behavior (i.e., when the animal inserts its head inside the hole), although qualitatively very different, form a stable cluster in untreated subjects (Casarrubea et al., 2009a) but highly sensitive to pharmacological manipulation of the animal’s anxiety condition (Casarrubea et al., 2009b, 2017). Therefore, the strong temporal link between these two behaviors is highly evocative of the rodent’s anxious state. From the half-matrix obtained *via* this procedure, researchers should create a dendrogram, i.e., a graphical tree representation that shows in a very simple and intuitive way the “*closeness*” between the various clusters of behaviors. A hypothetical dendrogram is presented in Figure 3. The S values of the half-matrix are shown in the ordinate. In practice, this Figure indicates that units A and B, based on the number of reciprocal transitions, are linked by a high S value, which is matched by the value of unit C, that occupies a more “peripheral” position. This illustrative approach has been fruitfully used in numerous papers (Espejo and Mir, 1993, 1994; Espejo et al., 1994; Casarrubea et al., 2008, 2009a,b, 2011a, 2012, 2015c, 2017). From a functional point of view, these analyses made it possible to categorize the different units of behavior into clusters, that is, “behavioral sets” in turn indicative of a more complex activity contextualized to the explored environment. For example, in the work of Espejo and Mir (1993) as well as in that of Casarrubea et al. (2006), three different clusters of behavioral units have been described with regard to rat behavior on the hot-plate: sniffing, primary noxious-evoked and escape responses. This categorization, highlighting the animals’ response to the nociceptive stimulus, allowed for a better interpretation of the rodent’s behavior in the specific experimental context. Importantly, these studies also demonstrated that these three categories are extremely sensitive to the administration of independent variables, such as, for example, specific drugs.

#### Probabilities of transitions

**Figure 4:**
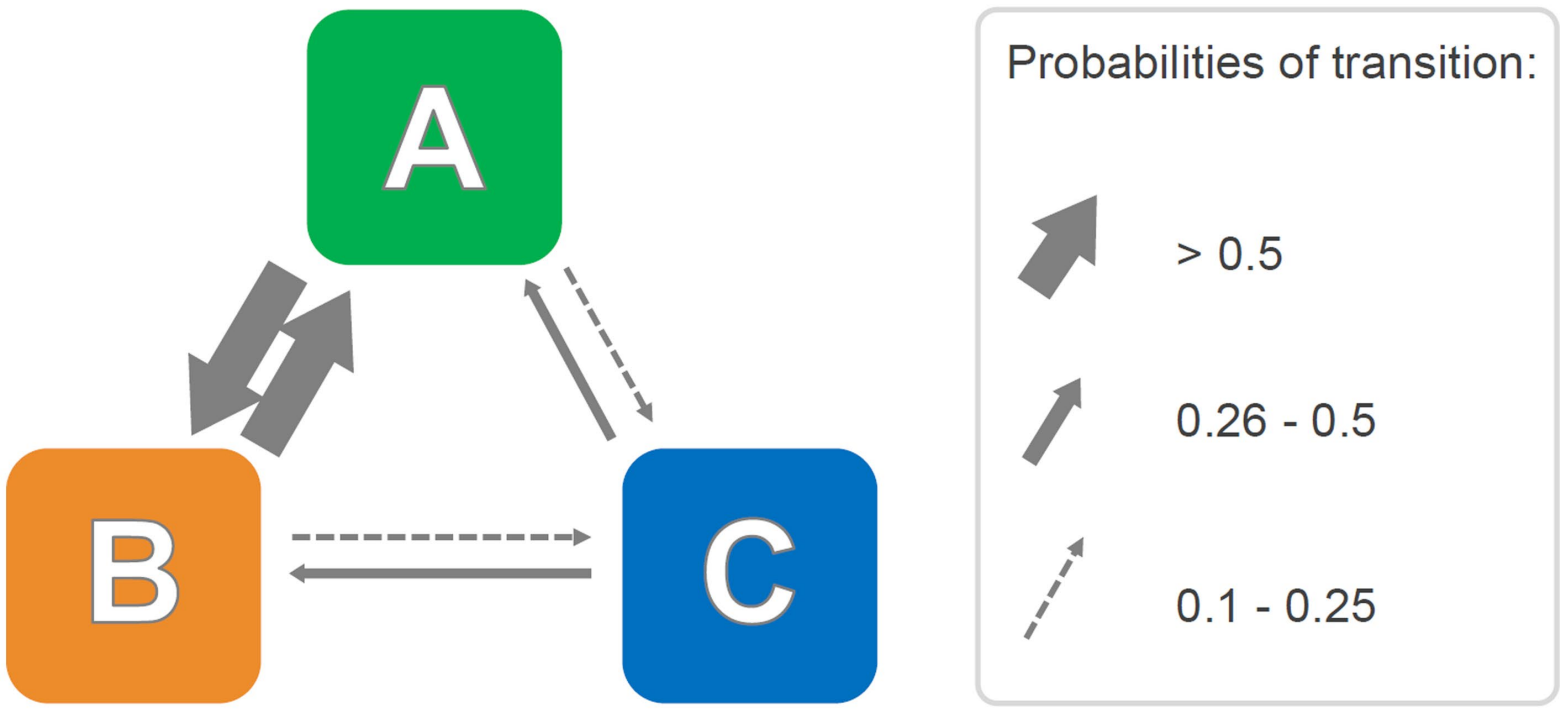
Hypothetical probabilistic pathway diagram representing transition probabilities among behavioral units. Right side, the selected probability ranges used to draw probability arrows of matching thickness. See text for details.

A stochastic approach is useful to emphasize probabilities of transitions among units of the behavioral repertoire. First, on the basis of relative frequencies of transitions among behavioral units, the transition matrix is transformed in a stochastic matrix. Following this step, the final matrix must meet three criteria: (1) for each row, the sum of all cells should be equal to 1; (2) the value of each cell should be between 0 and 1; and (3) the transition probability from one cell to the remaining cells should be equal to 1. A consistent advantage of such a matrix is that it can be graphically expressed through a pathway diagram, where transition probabilities among cells can be represented by means of arrows, with higher probability ranges showing as thicker arrows (Hemerik et al., 2006). Like the dendrograms briefly discussed in section 2.1., the use of probability diagrams is intuitive and emphasizes the temporal constraints between different behavioral units, providing a unified view of the behavioral repertoire. A hypothetical probabilistic pathway diagram is presented in Figure 4. In practice, it can be inferred from the Figure that units A and B share higher probabilities of mutual transitions than units B and C or units A and C. These probabilistic representations have been successfully used in several studies (Espejo and Mir, 1993; Espejo, 1997; Lino-de-Oliveira et al., 2005; Casarrubea et al., 2008, 2009a,b; Santangelo et al., 2018). Functionally speaking and similar to what was mentioned in the previous section regarding dendrograms, these analyses of the relationships among behavioral units in terms of probabilistic constraints allowed researchers to unravel otherwise unnoticeable behavioral dynamics characterizing subjects’ activity in the specific environment. For instance, Espejo (1997), in the Elevated Plus Maze, revealed the existence of a behavior heavily polarized toward sniffing episodes and the occurrence of two behavioral units (Stretched Attend Posture and Protected Dipping) sharing high reciprocal probabilities of transitions. The authors suggested that these two behavioral units may have a common behavioral significance in the form of “*anxiety-related behaviors, which mice usually display in succession and from relatively secure closed and central sections of the maze*” (Espejo, 1997, p. 109).

#### Lag sequential analyses

**Figure 5:**
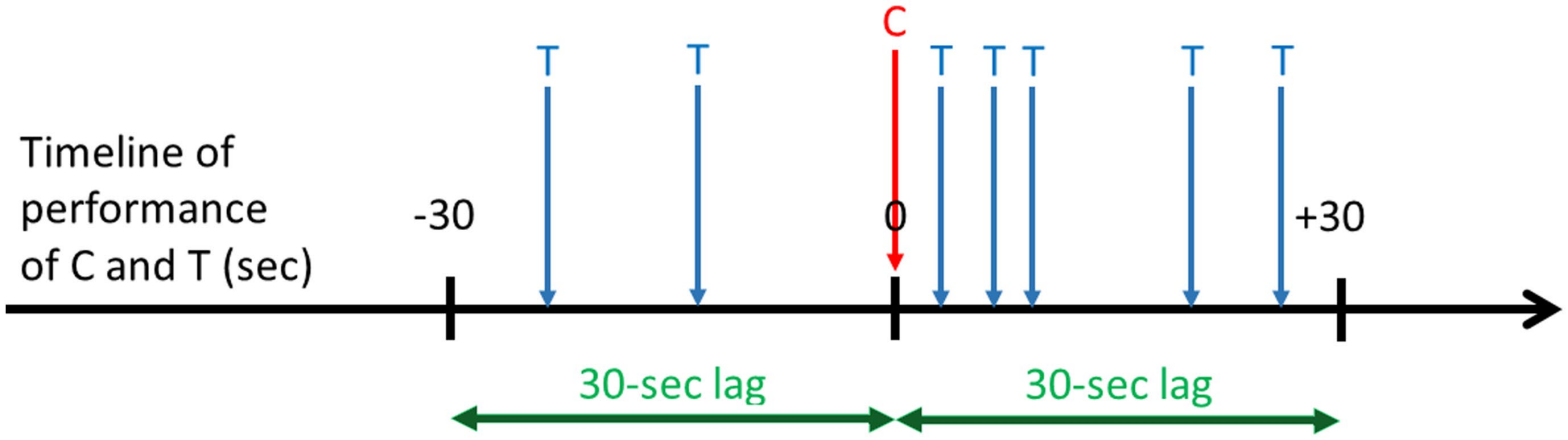
Hypothetical example of a 30-s time lag sequential analysis along a timeline of performance of a given Criterion behavior (C) in seconds. This analysis is used to compare the frequency of a Target behavior (T) within a 30-s window before and within a 30-s window after the start of C. For instance, T can be “being approached and being looked at by close neighbors.” This example indicates that T is more frequent shortly after the performance of C than shortly before.

**Figure 6:**
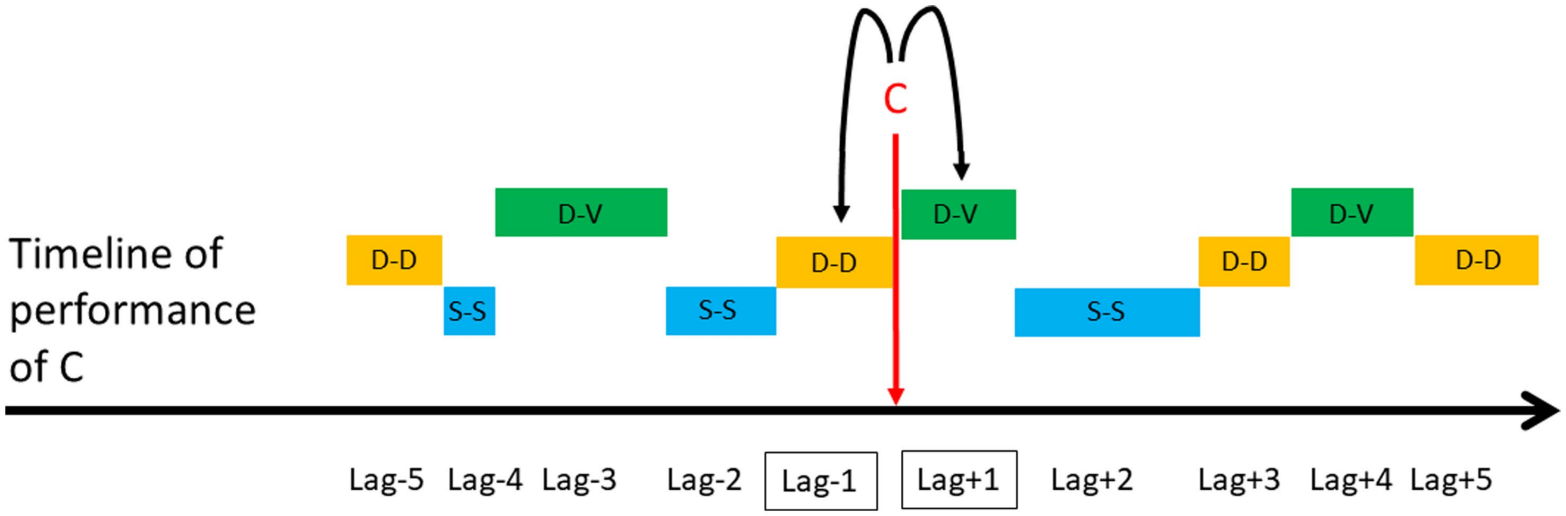
Hypothetical example of a state lag sequential analysis used to compare the frequency of a dorso-ventral (D-V) position (i.e., Target) adopted by the female consort partner immediately before (i.e., lag-1) versus immediately after (i.e., lag +1) the performance of a female-to-male mount (i.e., Criterion: C). Other positions relative to the male consort partner during intermount intervals include dorso-dorsal (D-D) and side-by-side (S-S). Other pairs of lag are lag +2 and lag-2, lag +3 and lag-3, etc. (Gunst et al. 2022).

Lag sequential analyses are an example of analytical techniques that rely on transition matrices (Sackett, 1979; Quera and Estany, 1984). LSA is a type of temporal analysis applied to behavioral sequences that calculates the frequency of transitions between pairs of behavioral units within a certain lag. The first event of the pair is called “Criterion” and the second “Target” (Faraone and Dorfman, 1987). Depending on which direction in time researchers are interested in investigating (i.e., positive or negative), they calculate how often the Criterion (e.g., Event A) was followed by the Target (e.g., Event B), or how often Target (e.g., Event B) preceded the Criterion (e.g., Event A), respectively. There are two types of LSA, depending on the type of transitions between a Criterion and a Target. First, a “time lag” sequential analysis requires the comparison of the same time window before and after the Criterion, and considers transitions between a Criterion and a Target within these specific time windows, independent of how many other events are between them. Researchers then calculate the number of transitions from a Criterion to those Targets occurring within a specific time window following or preceding the Criterion (Figure 5). Second, a “state lag” sequential analysis considers the transitions between a Target that directly followed or directly preceded a Criterion (i.e., lag +1 or lag-1, respectively). Other pairs of lag are possible, such as lag +2 or lag-2, lag +3 or lag-3, etc. (Figure 6). Among other examples, lag sequential analyses have been employed to investigate the motivational underpinnings and functional components of specific behavioral elements expressed within sequences of actions performed during playful or sexual activities in free-ranging macaques (Cenni et al., 2022; Gunst et al., 2022a,b, 2022).

#### Adjusted residuals

**Figure 7:**
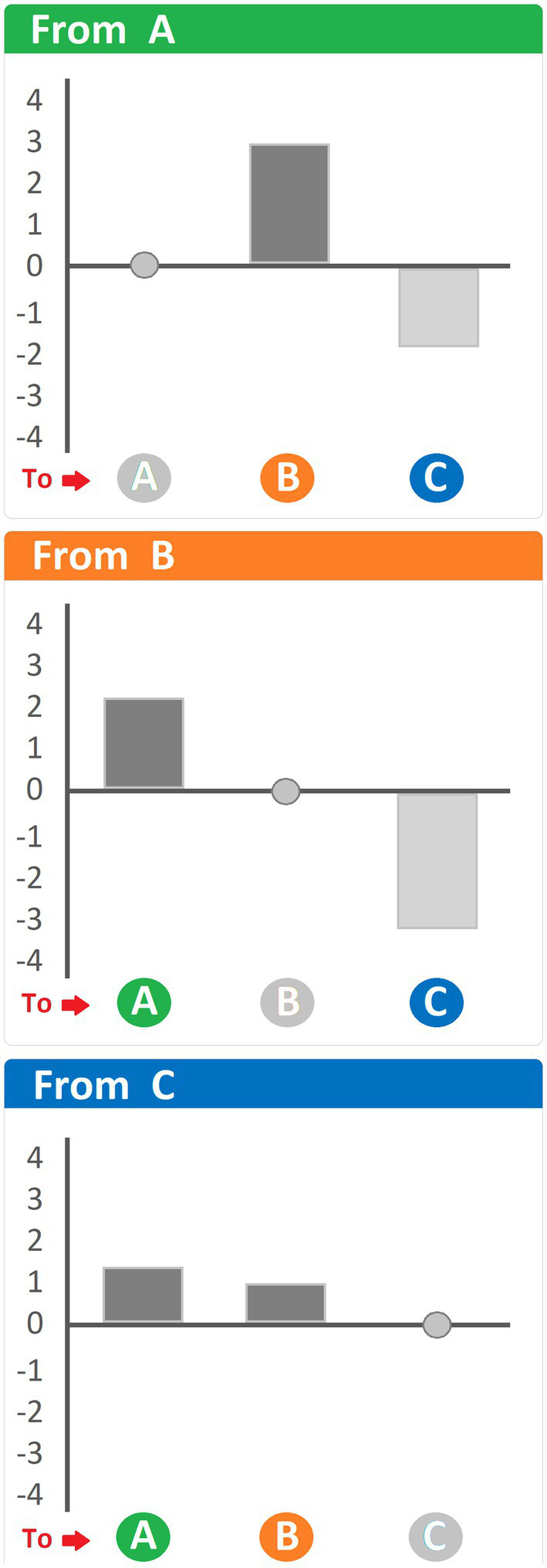
Hypothetical histogram of adjusted residuals representing the differences between observed and expected values. Heading of each panel: behavioral unit antecedent to the ones indicated at the bottom. Dark bars, positive residuals (i.e., transitions occurring more often than expected); light grey bars, negative residuals (i.e., transitions occurring less often than expected). See text for details.

One question that neither clustering procedures nor probability matrices can answer is the statistical significance of the transitions between behavioral units. To this effect, Haberman (1973) and Everitt (1977) proposed the conversion of a transition matrix into a matrix containing adjusted residuals. Adjusted residuals are standardized residuals divided by their respective standard deviations (Haberman, 1973; Everitt, 1977). Even though a specific formula is available to calculate residuals, a dedicated software allows for the automatization of this task when matrices reach hundreds cells. Various computer programs, such as Matman (Noldus Information Technology, Netherlands), make the calculation of adjusted residuals significantly faster and more reliable. The most important benefit of adjusted residuals is the possibility to utilize a common Z table for their interpretation: values ≥ +1.96 and ≤ −1.96 indicate statistically significant (*p* < 0.05) transitions occurring more often (≥ +1.96) or less often (≤ −1.96) than expected. Like probability matrices, matrices of adjusted residuals can be illustrated by means of pathway diagrams (Spruijt and Gispen, 1984; Spruijt, 1992; Vanderschuren et al., 1996; Van Den Berg et al., 1999), where the thickness of the arrows is also indicative of the significance level of the given transition. However, pathway diagrams of adjusted residuals come with a caveat. While representing positive residuals arrows is intuitive, representing negative residuals is problematic as negative residuals are transitions occurring significantly less often than expected, making their representation as arrows counter-intuitive. One solution, in this regard, is to present the residuals by means of positive and negative bars indicative of positive and negative residuals, respectively. A hypothetical representation of residuals using such an approach is shown in Figure 7. Examples of such an approach have been used in several studies (van Lier et al., 2003; Casarrubea et al., 2008, 2009a,b, 2012).

Adjusted residuals are extremely sensitive to changes in the transition matrix from which they derive, and their intrinsic statistical connotations make it possible to establish an important and useful “boundary” (that is, the significance level) to the various transitions that are taken into account or shown. For example, regardless of the illustrative approach used, one may therefore choose to present only those transitions that are statistically significant. This important aspect has been stressed by Spruijt and Gispen (1984) in a pioneering study going back almost four decades. In brief, this study showed the effects of ACTH on grooming behavior in rats and highlighted that observed changes should inform future interpretations of the animal’s behavior in the specific context in which it is observed.

### The temporal dimension

The temporal dimension adds an additional layer of complexity to the analysis of the possible relationships existing between different units of the behavioral repertoire. Indeed, such relationships may not be linear. What does it mean? In a sequence A➔B➔C➔D[…]➔W➔X➔Y➔Z, where each letter represents a behavioral event (*from a conceptual view, it is more correct to refer to units of behavior occurring over time in terms of “events”*), statistically significant relationships may not occur between events in direct succession (that is, between B and A, C and B etc.); actually, the opposite is almost always true and significantly related events are often not in direct succession (e.g., D and A, Y and W etc.). This makes temporal sequences difficult to be perceived and elusive to the naked eye. Eibl-Eibesfeldt (1970) himself emphasized this essential aspect by stating that investigations of behavior must deal with sequences that are not easily perceivable and this is why researchers need improved methods of detection and analysis of the temporal dimension of behavioral sequences.

The study of the temporal structure of behavior was, for many years, a difficult shore to reach mainly because of an unfortunate combination consisting of the lack of adequate computational resources compounded by the lack of efficient models to conduct these analyses. These difficulties have been gradually reduced over the past 30 years thanks to considerable technological development and the introduction of an elegant technique suitable for studying the temporal structure of behavior: the T-pattern analysis (TPA; Magnusson, 1996, 2000, 2004, 2005, 2017, 2020; Casarrubea et al., 2015a, 2018; Magnusson et al., 2016; Casarrubea and Di Giovanni, 2020). Through this approach, recurrent sequences of events, called T-patterns, can be easily highlighted and, therefore, studied. A remarkable aspect of the method is its complete independence from the underlying time scale. Indeed, TPA allows for the study of events that occur in the order of milliseconds (e.g., those related to the firing of neurons) as well as events that span much larger time windows (e.g., those related to rodent behavior). The detection of T-patterns can be performed by means of a software tool known as THEME (Patternvision Ltd., Reykjavik, Iceland).

**Figure 8:**
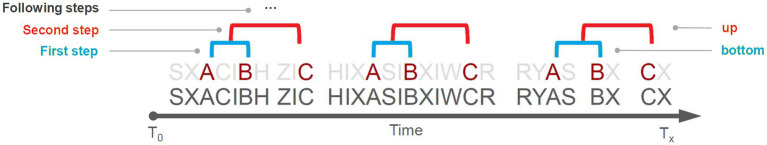
Hypothetical observation period (T0–Tx) consisting of 30 events (black letters near time axis). The [(A B) C] T-pattern becomes evident when all the remaining behavioral occurrences are left out (light grey letters). See text for details.

A T-pattern can be described using the following expression:


$$X_{1}\approx dt_{1}X_{2}\approx dt_{2}X_{3}\ldots X_{i}\approx dt_{i}X_{i+1}$$

where, X terms are the events belonging to a hypothetical T-pattern and ≈dt terms represent the time distances separating these events. Thus, the term X_1_ ≈ dt_1_ X_2_ indicates that the X_1_ event is followed dt_1_ time units later by the X_2_ event; so, X_i_ ≈ dt_i_ X_i + 1_ symbolizes that X_i_ is followed dt_i_ time units later by X_i + 1_. Figure 8 shows a sequence of 30 hypothetical events (letters near the axis) occurring in the context of an observation time window T_0_-T_x_. The detection algorithm compares the distribution of each pair of events (e.g., A and B) searching for an interval in which A is followed by B more often than expected by chance. If B does not fall within such an interval, another event (e.g., C) is tested, and so on, for all the events; if event B falls within the interval, A and B represent a T-pattern encompassing only two events and will be indicated as (A B). This (A B) T-pattern is then used by the algorithm to detect higher-order patterns, e.g., [(A B) C], [(A B) (C D)] etc. Such a bottom-up detection process runs up to any level and stops when no more T-patterns are identified. The use of TPA allows for the identification of three important qualitative aspects related to the temporal structure of behavior: its *variability*, *complexity* and *recursiveness* (Casarrubea et al., 2021b). The variability is represented by the amount of T-patterns of different composition detected, the complexity is the length of T-patterns (i.e., the number of events in their sequences), and the recursiveness is the number of times each T-pattern is repeated. More details concerning concepts, theories and procedures underlying the detection and analysis of T-patterns can be found in numerous papers of Magnusson (Magnusson, 1996, 2000, 2004, 2005, 2017, 2020; Magnusson et al., 2016) and in a number of works from our laboratory and field studies (Casarrubea et al., 2015a, 2018; Casarrubea and Di Giovanni, 2020; Cenni et al., 2020; Gunst et al., 2020). These studies have successfully shown that the temporal dimension of behavioral sequences represents a powerful structural variable to infer underlying behavioral and psychological mechanisms, and explain the actions being performed in terms of functional components.

## From rodents to non-human primates

Even though the above-outlined methods are not the only ones used to identify and describe the structural relationships between the different units of a behavioral repertoire, they are fruitful approaches. After providing examples of significant studies employing these analytical techniques across disciplines and animal taxa, we discuss the benefits and challenges associated with the use of these methods.

### Transition matrices and related analyses

Behavioral studies based on the analysis and transformation of transition matrices have produced a large number of publications straddling various scientific disciplines and including a wide range of animal taxa from insects (e.g., Berman et al., 2016) to reptiles (e.g., McElroy et al., 2012), fishes (e.g., Van Der Heijden et al., 1990), birds (e.g., Eens et al., 1989), rodents (e.g., Espejo and Mir, 1993), all the way to non-human primates (e.g., Skorupa, 1983) and humans (e.g., McCune and McCune, 2019). Despite the common use of transition matrices, these studies employ a great deal of different procedures, and it is beyond the scope of this article to explore the enormous variability in the application of these analytical methods. That said, within the research employing transition matrices the work conducted on the behavioral structure in rodents certainly stands out. This is not surprising, since mice and rats are, by far, the preferred species in biomedical research due to their relatively low cost, basic housing conditions, and availability of many genetic variants that are extremely useful from a translational point of view (Bryda, 2013). The approaches used in most of the articles in which transition matrices are used in the study of rodent behavior fall into one or more of the methods outlined above.

Among these studies, pioneering works include those by Spruijt and Colleagues, in which adjusted residuals are predominantly employed (Spruijt and Gispen, 1984; Spruijt, 1992; Vanderschuren et al., 1996; Van Den Berg et al., 1999) and those by Espejo and Colleagues, that often involves the simultaneous use of probabilistic evaluations and aggregative clustering techniques (Espejo and Mir, 1993, 1994; Espejo et al., 1994; Espejo, 1997). In the same vein, let us just mention a study by Lino-de-Oliveira et al. (2005) pertaining to locomotory behavior of rats subjected to the forced swimming test, an elegant study by Takahashi et al. (2010) on rats’ ultrasonic vocalizations, and numerous studies by Casarrubea and Colleagues focusing on the structure of exploratory behavior, anxiety-related response, and reaction to pain in rats and mice, and involving various lab apparatuses such an Open Field (e.g., Casarrubea et al., 2008; Santangelo et al., 2018), Hole Board (e.g., Casarrubea et al., 2009b, 2017) and Hot-Plate (e.g., Casarrubea et al., 2006, 2012).

In non-human primates, both “time lag” and “state lag” sequential analyses have been successfully used to demonstrate that female-to-male mounting in Japanese macaques is a supernormal courtship behavior that functions to focus the male consort partner’s attention and prompt subsequent male-to-female mounting (Gunst et al. 2022).

What are the strengths and weaknesses of the utilization of transition matrices? As far as benefits are concerned, whether the applications lie in a deep understanding of the structure of behavior in rodents or primates, these methods are unified by the possibility of presenting the results in an extremely direct and intuitive way, which makes the publications pleasant to read and the findings easy to understand for a wide and non-expert audience. One only has to look at the dendrogram (Figure 3), the pathway diagram (Figure 4) and/or the histogram of residuals (Figure 7) to immediately grasp the meaning of these graphical representations and how they relate to the original transition matrices, that would otherwise look obscure. Regarding which of these methods may better convey information about behavioral structure, probabilistic analysis and pathway diagrams make it more intuitive than the clustering procedure and/or dendrograms. However, both pathway diagrams and dendrograms require statistical support. This can be done by analyzing the individual transitions in the source matrices and adding the corresponding adjusted residuals that stand for the different transitions in statistical terms.

Let us now address some problems associated with using transition matrices. Unlike the simplistic graphical representations featured in Figures 3, 4, 7 that contain only three behavioral units, a typical transition matrix is a table containing dozens or even hundreds of numbers. Therefore, reading, analyzing, and interpreting a transition matrix is generally no simple matter not even an option for researchers accustomed to generating matrix analysis from data sets. Beyond the considerable work required to obtain the event log-files from which a transition matrix is derived (see section “Physiology of Behavior”), the matrix should then be subjected to specific transformations that direct the subsequent analysis toward clustering, probabilities, and/or residuals. Once this step is completed, the matrix is still an analytical item that only contains a large amount of numerical data and requires graphical treatment to be usable for interpretation (i.e., to obtain dendrograms, probability pathway diagrams, and histograms with residuals). In other words, one of the main problems associated with the use of transition matrices is the enormous amount of time required for their processing, analysis, and graphical transformation to make them intelligible. Yet, there is an even greater problem. The dendrograms, pathway diagrams and histograms containing positive and negative residuals are analogous to photographs with a very long exposure: on the one hand, they describe the entire observation period but, on the other hand, they lack one essential feature for any behavior: its temporal dimension. As pointed out by Eibl-Eibesfeldt (1970), behavior is structured on the basis of a flow of events that runs through time.

### Temporal dynamics of behavior

Over the past three decades or so, the search and analysis of T-patterns have been successfully and widely used by scientists from a number of different fields, including psychology, psychiatry, computer science, physiology, biology, and sport science. The reasons underlying the inter-disciplinary popularity of TPA is that this technique offers researchers a great level of detail in the temporal structure of the behaviors under study. TPA has been successfully employed to explore the temporal organization of behavior in numerous species, ranging from rodents to primates.

In rodents, TPA allowed researchers to describe the qualitative and quantitative aspects of feeding behavior in rats under different dietary regimes (Casarrubea et al., 2019a), analyze anxiety-related behaviors in different rat strains (Casarrubea et al., 2010, 2011b, 2013a,b, 2014, 2015b, 2016, 2020a, 2021a), examine psychostimulant-evoked route-tracing stereotypies in mice (Bonasera et al., 2008), as well as generate a model of Tourette’s syndrome (Santangelo et al., 2018) and a model of Parkinson’s Disease (Casarrubea et al., 2019b) in rats.

In non-human primates, TPA has recently been used to demonstrate that the temporal dynamics of a behavior is informative to infer its function, and this, in two different behavioral domains: tool use (Cenni et al., 2020) and non-conceptive sex (Gunst et al., 2020). First, in a free-ranging population of Balinese long-tailed macaques in which versatile stone play was identified as a behavioral tradition, the monkeys were reported to repeatedly tap and rub stones onto their genital area (Pelletier et al., 2017). Despite those two stone-directed actions being integrated into stone play episodes, TPA revealed that the temporal structure of stone play sequences with genital stone-tapping and genital stone-rubbing performed by males was less structurally flexible and less exaggerated than that of stone play sequences without genital stone-tapping and genital stone-rubbing, suggesting functional attributes of these two specific behavioral units (Cenni et al., 2020). Thus, the performance of genital stone-tapping and genital stone-rubbing by male Balinese long-tailed macaques could be an example of stone play actions being functionally recycled into stone tool-assisted masturbation (Cenni et al., 2020). This result supports the view that object play can serve as a pool of behavioral variability and has the exaptive potential to be subsequently co-opted into tool use (Leca and Gunst, in press). Second, in a free-ranging population of Japanese macaques in which female-to-male mounting was identified as a culturally-maintained form of non-conceptive sex, TPA showed that the occurrence of female-to-male mounting conferred further functional constraints to mating sequences with more hierarchically organized and less repeatable courtship behaviors than in mating sequences without female-to-male mounting (Gunst et al., 2020). This result supports the view that female-to-male mounting in Japanese macaques is a supernormal courtship display more efficient than species-typical female-to-male sexual solicitations at prompting subsequent male-to-female mounts (Gunst et al. 2022).

In humans, TPA has been widely applied to detect tactical movements or prevent injuries in several sports and physical activities, such as boxing (Pic and Jonsson, 2021), taekwondo (Gutiérrez-Santiago et al., 2020), and football (Prieto-Lage et al., 2020), as well as to explore the relationships between impulsivity and physical activity (Castañer et al., 2020). TPA was also used in the study of human-animal interactions (Kerepesi et al., 2005), human-robot interactions (Kerepesi et al., 2006), hormones and behavior (Hirschenhauser et al., 2002), decision-making processes (Pic et al., 2021), eye-blinking behavior (Brill and Schwab, 2020), movement and behavioral disorders (Aiello et al., 2020), neuropsychiatric diseases (Lyon and Kemp, 2004; Kemp et al., 2008, 2016; Sandman et al., 2012) and the behavior of preschool-age children (Santoyo et al., 2020).

The first advantage of using TPA in behavioral research is directly related to the outputs of this approach: providing detailed information, otherwise impossible to access, about the temporal structure of behavior. To the best of our knowledge, no other analytical techniques allow for the study of the temporal dynamics of behavior at such a fine-grained resolution. Another benefit of TPA is its independence from the observed time window; whether it is a few seconds (typical of neuron firing measurements), a few minutes (typical of experimentally-induced behavioral measurements in lab rodents), or hours (typical of the assessment of a daily activity budget in free-ranging primates), the principles of T-pattern detection remain the same. Finally, TPA allows researchers to obtain three essential qualitative characteristics of the temporal texture of behavior, namely its variability, complexity, and recursiveness (Casarrubea et al., 2019a, 2021b). The evaluation of such qualitative aspects, combined with the possibility to analyze specific events in the structure of the identified sequences allow for a further understanding of the behavior. For example, comparisons between rats treated with standard diet and rats treated with hyperglycidic diet revealed profound variations in those qualitative parameters. Specifically, animals under standard diet showed a behavior characterized by fewer T-patterns of different composition (i.e., lower temporal variability) which are more often repeated (i.e., higher recursiveness); on the other hand, rats under a hyperglycidic diet showed a behavior characterized by a noticeably higher number of different T-patterns (i.e., higher temporal variability) but that are repeated less often (i.e., lower recursiveness). The evaluation of the qualitative aspects of these patterns showed a significantly higher percentage of T-patterns specifically associated with two behavioral units, the so called Focusing-Sniffing (i.e., when the rat sniffs the rim of the pellet box without inserting the head inside) and Feeding (i.e., when the rat inserts the head inside the pellet box and eats) in the hyperglicidic group than in the control group. These results allowed researchers to hypothesize an increased salience of food-related stimuli in rats under hyperglycidic diet and a behavior highly evocative of craving (Casarrubea et al., 2019a).

One of the main challenges associated with the use of TPA is to fine-tune the use of the software with the data at hand. The most recent versions of the software tool utilized to perform T-patterns’ detection analysis do feature numerous options; a thorough knowledge and practice of each of them is crucial to the reliability and validity of any outputs. Conversely, poor use of the software parameters can lead to the detections of T-patterns that might not be salient to the research question or difficult to interpret. An additional set of difficulties pertaining to the use of TPA is common with the analysis of transition matrices. TPA software also require event log-files to perform T-pattern detection. The creation and organization of such log-files before import into the software tool, albeit not complex in theory, are extremely time-consuming tasks (see section Physiology of behavior.). Although we often speak of “T-pattern analysis” to refer to the entire analytical process, this essential preparatory step should be kept in mind.

## Conclusion

In this paper, we aimed to present the main methods that have accompanied us in the structural analysis of behavior over the past two decades. Our goal was not to present an excursus on the use of multivariate analyses in this line of behavioral research. Instead, we sought to provide some theoretical background for beginners in the exploration of behavioral structure and for already experienced researchers who wish to implement some of the approaches outlined here. Structural behavioral analyses provide substantial benefits that, by far, compensate for the large amount of time required for their use. The main advantage of these methods consists in producing qualitative and quantitative descriptions of complex behavioral dynamics that would otherwise be difficult or impossible to obtain.

## Author contributions

MC conceived the study. J-BL, NG, GJ, GC, and MC wrote the manuscript. MC, J-BL, NG, and SA conducted field-and laboratory-based research and performed data analysis. MC prepared tables and Figures. GC, GG, J-BL, NG, GJ, and MP reviewed and edited the manuscript. All authors contributed to the article and approved the submitted version.

## Funding

J-BL’s research was funded by Natural Sciences and Engineering Research Council of Canada (NSERC, Discovery Grant #: 2015-06034 to J-BL). MC, SA, and GC’s research was funded by a grant from the University of Palermo, Italy.

## Conflict of interest

The authors declare that the research was conducted in the absence of any commercial or financial relationships that could be construed as a potential conflict of interest.The reviewer MB declared a past co-authorship with the authors MC and GG to the handling Editor.

## Publisher’s note

All claims expressed in this article are solely those of the authors and do not necessarily represent those of their affiliated organizations, or those of the publisher, the editors and the reviewers. Any product that may be evaluated in this article, or claim that may be made by its manufacturer, is not guaranteed or endorsed by the publisher.
